# Suicide, Stigma and COVID-19: A Call for Action From Low and Middle Income Countries

**DOI:** 10.3389/fpsyt.2022.894524

**Published:** 2022-07-01

**Authors:** Sheikh Shoib, Miyuru Chandradasa, Fahimeh Saeed, Aishatu Yusha’u Armiya’u, Thiago Henrique Roza, Dorottya Ori, Jitender Jakhar, Nuno Rodrigues-Silva, Debanjan Banerjee

**Affiliations:** ^1^Department of Psychiatry, Jawahar Lal Nehru Memorial Hospital, Kashmir, India; ^2^Department of Psychiatry, University of Kelaniya, Ragama, Sri Lanka; ^3^Department of Psychiatry, Psychosis Research Center, University of Social Welfare and Rehabilitation Sciences, Tehran, Iran; ^4^Department of Psychiatry, College of Medical Sciences, Abubakar Tafawa Balewa University, Bauchi, Nigeria; ^5^Department of Psychiatry, Federal University of Rio Grande do Sul, Porto Alegre, Brazil; ^6^Department of Mental Health, Heim Pal National Pediatric Institute, Budapest, Hungary; ^7^Institute of Behavioural Sciences, Semmelweis University, Budapest, Hungary; ^8^Fortis Hospital, New Delhi, India; ^9^Mental Health Unit, Hospital School of the University Fernando Pessoa, Gondomar, Portugal; ^10^Faculty of Health Sciences, University of Beira Interior, Covilhã, Portugal; ^11^Consultant Geriatric Psychiatrist, APOLLO Multispecialty Hospitals, Kolkata, India

**Keywords:** suicide, stigma, COVID-19, mental health, psychiatry

## Abstract

Suicide is a global health issue that needs to be addressed. The COVID-19 pandemic has resulted in an increased mental health burden. Stigma has obstructed efforts to prevent suicide as individuals who need urgent support do not seek appropriate help. The influence of stigma is likely to grow in tandem with the COVID-19 pandemic. The stigmatization of persons with mental illnesses is widespread worldwide, and it has substantial effects on both the individual and society. Our viewpoints aim to address the probable link between stigma and suicide in the wake of the current pandemic and propose ideas for reducing suicide-related stigma.

## Introduction

Suicide is one of the leading causes of death globally, with this trend being more pronounced in younger people and low and middle income countries (LMIC). According to the World Health Organisation (WHO), over 77% of suicide deaths worldwide occur in LMIC, with more than 700,000 suicide deaths reported in 2019 and 173,347 in India alone (1). Suicide is thus considered a global public health concern.

COVID-19 pandemic was the most pressing issue faced in the 2020–2022 period, and it had a detrimental effect on communities, including patients and healthcare workers. Pandemics are not just medical experiences; they cause interference in nearly all biopsychosocial dimensions. According to the WHO, until May 18, 2022, there have been approximately 523 million confirmed cases of COVID-19, and the disease has caused the death of more than 6.27 million people worldwide. LMICs have suffered more intensely with the pandemic, accounting for many cases and deaths. For instance, the death count in Brazil surpassed 665,000 and 325,000 in Mexico; in India, the number is approximately 520,000, and in Iran, more than 141,000 have died due to COVID-19 (2). In addition to the impact of the virus on the mental health of affected populations, social distancing, quarantine and other similar measures used to control the spread of the virus also imposed significant negative psychosocial consequences such as post-traumatic stress disorder and depression (3).

The mental health burden has increased due to the COVID-19 pandemic, and communities worldwide require additional psychosocial support. This paper highlights the stigma and discrimination associated with suicides in COVID-19 and how it plays a core role in managing suicidal risk in the community.

### COVID-19: The Potential Risk of Suicide

There is debate about the increase in suicidal ideations after the beginning of COVID-19. A multicenter study in 21 countries reported that the numbers have remained unchanged or declined in the early pandemic compared to the pre-pandemic period in high-income and upper-middle-income countries (4). According to a systematic review of many studies about suicidal behavior related to the COVID-19 pandemic from November 2019 to September 2020 that included 120,076 persons, there has been an increase in suicidal ideation rates in comparison with the period before the pandemic, with a pooled prevalence of approximately 12% (5). The same study described several risk factors associated with suicidal ideation during the pandemic, including quarantine, loneliness, sleep issues, poor social support, and mental and physical exhaustion (5). Social distancing and subsequent social isolation, economic problems due to lockdown policies and unemployment, social stigma and discrimination, fear of the virus, stress, and the burden of work that some professionals have experienced have also been pointed out as potential factors associated with suicide during the COVID-19 pandemic (6). Certain vulnerable groups are at an increased risk of suicide and other self-harm related behavior, such as the elderly, persons with mental disorders, healthcare professionals, the homeless, and migrant workers (6). During the pandemic, they should be the target of specific preventive measures by the services.

### Stigma and Suicide

Stigma is a profoundly discrediting attribute and encompasses several components, i.e., labeling, stereotyping, separation, status loss, and discrimination (7, 8). Stigma can also be classified into public stigma and self-stigma (9). The former refers to the general population’s reaction toward people with devalued characteristics, while the latter occurs when people with these devalued characteristics endorse the public attitudes and experience the negative consequences themselves. Stigma has been shown to display deleterious effects; for example, it has been shown to harm the self-esteem of those with mental illness (10). Public stigma may lead to social rejection, which leads to inequality in employment, access to health care and social participation (11). Stigma may be reduced by three approaches, i.e., protesting, education, and avoiding isolation (12). By protesting against inaccurate information held toward the devalued individuals, educating with the correct information and increasing contact between the public and the devalued individuals, the stigma is likely to diminish in certain settings.

The stigmatization of people with mental disorders is prevalent worldwide and leads to severe consequences for both the individual and broader society. Stigma in itself has a bidirectional relationship with mental illness and suicidality. Furthermore, patients with mental illnesses often face stigmatization, while on the other hand, stigma can precipitate mental illness and suicide (13). In addition, suicide is associated with stigma, which can manifest not only in suicide survivors but also in family members and close friends of victims of suicide. Thus, stigma may bring an additional burden to an already distressed individual, imposing stereotypes, distrust, a bad reputation, and a mark of disgrace, which can be produced by external members of the society or by the person her/himself (14). Suicide has also been equated with crime, punishment or sin, perpetuating stigma and impairing help-seeking (15). Stigma can lead to demoralization, feelings of isolation, loneliness, and hopelessness, leading to an increased risk of suicide (16). Also, legislation in certain nations criminalizing suicide is responsible for increasing stigma. Considering that stigma is also associated with ignorance and negative attitudes toward a specific phenomenon, it is essential to educate the public about suicide, associated factors, treatment and preventive strategies, and stimulate seeking care for those in need during the COVID-19 pandemic (16). It has been observed that financial crisis, unemployment, and poverty are the most prominent risk factors for suicide during the COVID-19 pandemic (17, 18).

### COVID-19 and Associated Stigma

Isolation, physical distancing, lockdown and unemployment can be very demanding. COVID-19 has been an “infodemic” where misinformation and fake news have led to fear and stigma that add to the current crisis (19). There have been concerns about stigma and discrimination in previous pandemics, and COVID-19 is no exception. Multiple reasons have been attributed, such as improper information about the spread of disease and increased fear and anxiety. This is further aggravated by measures like isolation and physical distancing, which are essential for preventing the spread of disease. The risk of losing a loved one can be extrapolated to social and moral circumstances, which can further cause stigma. However, the actual origin of stigma is complex and may extend beyond concepts such as social disability or moral transgressions (20).

Public health emergencies, such as this pandemic, are stressful for people and communities. Fear and anxiety about the disease could lead to social stigma, labeling, stereotyping, discrimination and other negative behaviors toward others. For example, stigma and discrimination can occur when people link a disease, such as COVID-19, with a population, community, or ethnicity (21). Stigma can also happen after a person has recovered from COVID-19 or been released from home isolation or quarantine. Identified factors related to stigma in the pandemic have been listed in Table 1.

**TABLE 1:** Aspects that influenced increased stigma related to COVID-19.

Belonging to an ethnic or racial minority. E.g., in the United States, Asian Americans (21), people of Northeast India (40)
Who has tested positive for COVID-19 or were released from quarantine (41)
Gender and sexual minorities experience more significant disparities (42)
Emergency responders or healthcare providers (43)
Essential workers, such as delivery drivers (44)
Older persons, especially seniors living alone in care homes (45)
People with a disability or physical impairment (46)
People experiencing homelessness (47)

### Heightened Stigma Leading to Suicidal Behavior

The social and economic hardships during this pandemic have negatively affected psychological wellbeing. The pandemic has been thought to have increased the risk of suicide among the frontline workers, elderly, migrants, homeless, poor, persons with mental disorders, and substance use disorders (22). The fear of COVID-19 infection, social boycott and loneliness during the quarantine were significant risk factors for suicidal behavior (23). Stigma leads to social isolation and discriminatory behaviors and undermines social cohesion in society, limiting opportunities for social interaction. Durkheim’s theory suggests that a breach in an individual relationship with society is a significant risk factor for suicide, and social integration has an inverse relation with the suicide rate (24). A study assessing the role of stigma in suicidal behaviors related to the interpersonal theory reported an indirect relationship between stigma to suicide-related perceptions (25). Further, the perceived burdensomeness and felt stigma contribute to suicide risk in vulnerable individuals (25).

**FIGURE 1:**
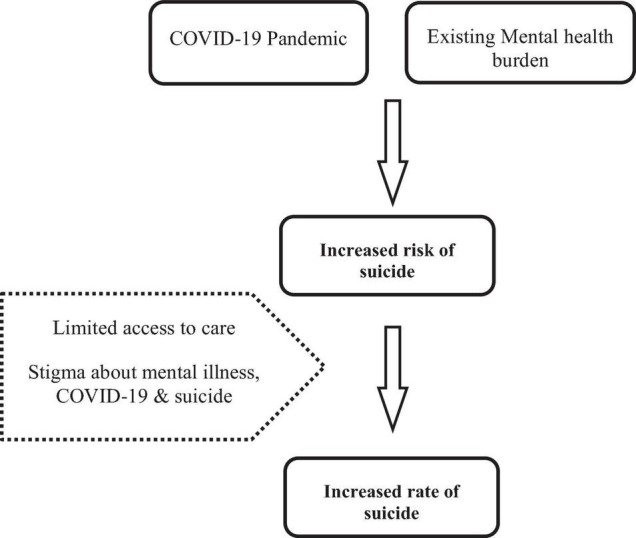
Relationship between COVID-19 pandemic, stigma and risk of suicide.

Stigma instills feelings of hopelessness, loneliness, anxiety, and anger in people who have experienced it, making them more prone to self-harming behaviors. Due to the COVID-19 pandemic, patients with recognized psychiatric problems might be unable to take their medications, causing symptoms to worsen and an increased risk of self-harm (26). Domestic and intimate partner violence has escalated due to the pandemic, resulting in psychological suffering and thoughts of self-harm among vulnerable couples (27). A recent study in Bangladesh reported that several factors, including the death of family members due to COVID-19, financial distress, domestic violence, alcohol consumption, social isolation, inaccurate information, stigma, and pandemic-related fear, ignited fear of suicidality (28). Figure 1 shows a proposed relationship between stigma and suicide during the COVID-19 pandemic. Since suicide is a complex and multidimensional problem, recognition and knowledge from various approaches help us explore the problem and contribute to meaningful intervention strategies (29).

### Empirical Findings on Stigma During the COVID-19 Pandemic

An adapted version of the Chronic Illness Anticipated Stigma Scale found that those who anticipated higher COVID-19 stigma and endorsed COVID-19 stereotypes to a greater degree would be less likely to seek a COVID-19 test (30). The Social Impact Scale (SIS), a widely used 24-item measure of stigmatization used for patients with medical conditions and infectious diseases such as HIV, has been used (31). It was found that COVID-19 patients experienced stigma, social rejection, financial insecurity, internalized shame and social isolation. They also showed that depressive symptoms were positively associated with overall stigma levels (31).

### Reducing Stigma

Given these deleterious effects of stigma, more actions should be implemented to reduce the effect of stigma on individuals with suicidal behavior, enabling them to seek help. To date, evidence shows that mass media campaigns and reporting of suicide can modify beliefs and attitudes toward suicide (32). On the other hand, interventions including social contact and education effectively reduce stigma, which would hopefully reduce the risk of suicide (33).

Since the psychiatrists have a significant role in providing mental health services, early-career psychiatrists (E) are in the frontline; they play leadership roles in health care development and suicide prevention (34). They have first-hand experience observing the deleterious impact of stigma on a suicidal individual, from help-seeking to treatment completion. Moreover, since the beginning of the pandemic, trainees and young specialists have been regularly redeployed to critical care units or COVID wards in addition to their work on psychiatric services. Suicide prevention training in ECPs, especially in the COVID-19 crisis, would add to the mental healthcare delivery package (35). ECPs are uniquely positioned to bring together knowledge regarding the pathophysiology and epidemiology of COVID-19 as medical doctors, a sound understanding of suicide and other mental health issues, and a comprehensive approach to stigma as psychiatrists, with an active, clear, and straightforward approach and informative presence in the social media (36).

Therefore, ECPs need to have a prominent role in the fight against COVID-19 and suicide-related stigma, informing, teaching, promoting research, and influencing public policies (37). This can serve as an integral suicide prevention approach during the present crisis, subject to further research. Moreover, ECPs can help screen for mental health conditions in at-risk individuals, such as recovered individuals, health care workers, and close contacts.

Organizing support networks for at-risk individuals may provide powerful stress-buffering effects at an interpersonal level (38). Educating frontline staff who care for individuals with COVID-19 about stigma and providing care and support to the staff may also help reduce stigma and its associated effects such as burnout and work stress. At the community level, ECPs should adopt educational approaches to debunk unscientific beliefs surrounding COVID-19 and to publicize the nature of stigma on COVID-19, mental illness and suicide through mass media campaigns. Activities such as giving a voice to stigmatized COVID-19 survivors will enhance the public understanding of the impact of stigma, reducing discrimination by the general public. Advocacy interventions to seek support and recognition from policy developers on measures to minimize inequalities faced by the stigmatized individuals are essential. ECPs should research the complex relationship between COVID-19 related stigma and suicide and provide more scientific evidence to implement interventions. Lived experiences of suicide survivors, stigmatized frontline health workers, and ECPs themselves matter to shape health and policy interventions (39).

### Intervention

Finally, targeted interventions and a collaborative approach are required at various levels: individual, community and governmental. Suggested recommendations at each level are provided below in Table 2. By implementing these strategies, COVID-19 and suicide-related stigma could be addressed, reducing suicide risk in the community.

**TABLE 2:** Recommendations to reduce the stigma associated with suicide in the context of COVID-19.

Individual
Targeting the vulnerable groups such as frontline workers, people affected by COVID-19, sexual minorities, and migrant workers during the ongoing pandemic
Focusing on accurate personal communications, correcting negative language that can cause stigma, and sharing reliable information with contacts and on social media
**Community**
Promoting non-judgmental and open communication with suicide survivors and their families
Monitoring misinformation related to suicide on digital and social media platforms and reporting them
Reporting hateful online content about suicides, victims, and survivors to host platforms
Checking images used in the media for health promotion showing diverse communities and does not reinforce stereotypes
Enforcing ethical reporting of suicide deaths with details of psychological support for readers
Community awareness campaigns such as infographics, street plays, online educational videos, skits, and debates about the prevention of suicides
**Governmental**
Maintaining policies and guidelines ensuring the privacy and confidentiality of those seeking help for mental health issues
Introducing state-wide risk assessment protocols and validated tools for screening risk to self
Advocating for the rights of individuals living with mental illness and other vulnerable groups
Thanking healthcare workers, responders, and others working on the frontline will significantly encourage them
National and international collaboration among the ECPs to promote research and disseminate evidence-based guidelines in mitigating suicide-related stigma
Structured programs to screen and manage common psychological disorders
Allocating funds for public mental health promotion focusing on suicide prevention, victims, and grieving families

## Data Availability Statement

The original contributions presented in the study are included in the article/supplementary material, further inquiries can be directed to the corresponding author/s.

## Author Contributions

SS conceptualized and wrote the first draft. All other authors contributed equally to critical revision and writing of the final version.

## Conflict of Interest

The authors declare that the research was conducted in the absence of any commercial or financial relationships that could be construed as a potential conflict of interest.

## Publisher’s Note

All claims expressed in this article are solely those of the authors and do not necessarily represent those of their affiliated organizations, or those of the publisher, the editors and the reviewers. Any product that may be evaluated in this article, or claim that may be made by its manufacturer, is not guaranteed or endorsed by the publisher.
